# Mode of delivery and maternal outcome in subsequent delivery after an obstetric anal sphincter injury: a Finnish retrospective cohort study

**DOI:** 10.1186/s12884-025-07882-9

**Published:** 2025-07-19

**Authors:** Elina Ristilä, Outi Palomäki, Heini Huhtala, Elli Toivonen

**Affiliations:** 1 Center for Child, Adolescent and Maternal Health Research, Faculty of Medicine and Health Technology, University of Tampere, Tampere, Finland; 2 Department of Obstetrics and Gynecology, Tampere University Hospital, The Wellbeing Services County of Pirkanmaa, Tampere, Finland; 3 Faculty of Social Sciences, University of Tampere, Tampere, Finland

**Keywords:** Obstetrical anal sphincter injury, Third degree tear, Fourth degree tear, Subsequent birth, Vaginal delivery, Cesarean section

## Abstract

**Background:**

Obstetric anal sphincter injury (OASI) is a severe complication of vaginal delivery. In previous studies, parturients with a preceding OASI are at increased risk of a recurrent OASI (rOASI) in subsequent vaginal deliveries. In Finland, the rate of OASI is low compared to other countries, at 1.4% of deliveries, and the incidence of rOASI is not well-known. This study examined recurrence and mode of delivery after an OASI.

**Methods:**

This historical cohort study includes 278 women who have experienced an OASI and have delivered again in Tampere University Hospital. Deliveries complicated by an rOASI were compared to those without an rOASI, and women planning a cesarean delivery (CD) for their subsequent delivery were compared to women planning a vaginal delivery. Risk factors for OASI were explored by comparing deliveries complicated by an OASI to all deliveries.

**Results:**

After an OASI, 78.1% of parturients planned a vaginal delivery and 21.9% a cesarean delivery (CD). Vaginal delivery was successful in 98.1% of cases and only 1.9% of parturients who underwent vaginal delivery experienced an rOASI. Due to the low incidence rate, no risk factors for rOASI could be identified.

Parturients were most likely to have a CD in their subsequent delivery when the delivery complicated by an OASI was induced, the second stage was prolonged, episiotomy was performed, or the delivery had been assisted. The most common indication for CD was maternal request or fear of childbirth (85.9%). Assisted vaginal delivery, birthweight > 4,000 g, episiotomy, and postterm pregnancy were more common in deliveries complicated by OASI compared to all other vaginal deliveries in the study hospital during the same time period.

**Conclusions:**

The recurrence rate of OASI was low and the vaginal uncomplicated delivery rate was high among women who chose it for their subsequent delivery after an OASI.

## Key message

In a population with low primary incidence of obstetric anal sphincter injury, the rate of recurrent injury in subsequent vaginal delivery was low (1.9%). After counseling, vaginal delivery is also an option for women with a preceding obstetric anal sphincter injury.

## Introduction

Obstetric anal sphincter injury (OASI) occurs in about 1.4% of vaginal births in Finland [1] the incidence being among the lowest in Western countries, although even lower numbers have been reported [2, 3]. Despite appropriate diagnosis and care, OASI can cause prolonged symptoms such as anal incontinence, pain and sexual dysfunction [4, 5], highlighting the importance of preventing the injury. The low incidence of OASI in Finland has been attributed to the Finnish midwives’ tradition of strongly supporting the perineum at the time of expulsion of the fetal head (the “Finnish grip”), which has been routinely taught in Finnish midwifery training for many decades. The technique entails applying controlled resistance to the expulsion of the fetal head while concurrently approximating the perineal tissue toward the midline [6]. In Norway, introducing this maneuver has decreased the incidence of OASI [7]. 

Parturients with a previous OASI must consider which mode of delivery is the safest option and have to balance the risks associated with cesarean delivery (CD) against those associated with recurrent OASI (rOASI) [8–10]. The risk of OASI is known to be higher with primiparous parturients [11]. The other factors associated with increased risk of OASI (such as short stature, presentations other than occiput anterior, instrumental vaginal delivery or shoulder dystocia) are in turn not related to parity or are even more common in multiparous parturients, like advanced maternal age and increasing birth weight [2, 11, 12]. The risk of OASI in the subsequent birth has been reported to be significantly higher in parturients who suffered an OASI in the previous birth compared to multiparas in general [11]. In a Nordic register-based study, a rOASI was diagnosed in 2.1% of subsequent deliveries following an OASI in Finland, with a high elective CD rate (47%) [13]. Data on rOASI is scarce due to the low incidence of OASI in the study population. At our hospital, elective CD is recommended only for standard obstetric indications in subsequent deliveries following an OASI. However, elective CD can be performed upon maternal request. An estimation of fetal weight is performed in late pregnancy, and the management of labor onset tends to be conservative, favoring spontaneous labor unless complications such as gestational diabetes combined with macrosomia are identified. We hypothesized that the rate of rOASI would remain low even with lower CD rates.

The main objective of this study was to investigate the mode of delivery and risk of rOASI in parturients with a preceding delivery complicated by an OASI. The prespecified secondary aims were to assess factors affecting the choice of delivery mode in subsequent pregnancy and to identify risk factors for rOASI.

## Materials and methods

**Fig. 1:**
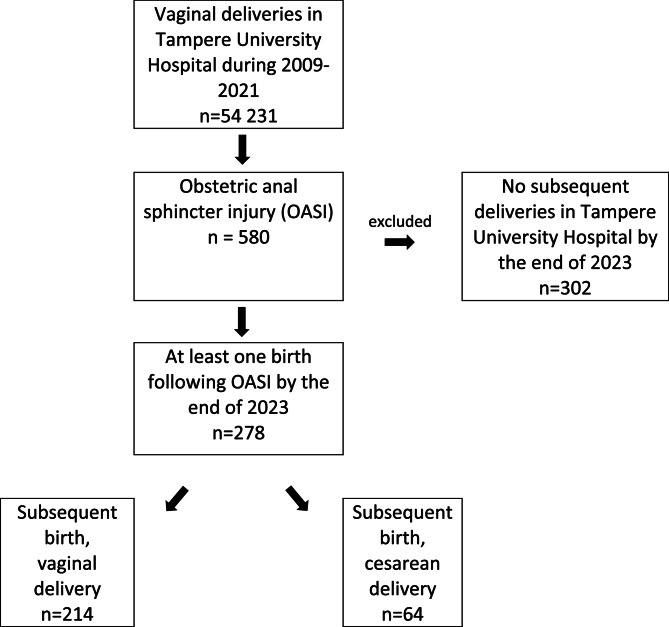
Flowchart. Mode of delivery in pregnancies following obstetric anal sphincter injury (OASI). Of the included parturients, 258 were primiparous at the time of OASI, whereas 8 had their first vaginal delivery after a cesarean delivery

This is an observational cohort study including all parturients who experienced an OASI between 2009 and 2021 and had at least one subsequent pregnancy leading to delivery in Tampere University Hospital by the end of 2023. Patients with an OASI were identified with ICD-10 codes O70.2 and O70.3, and only those who had given birth again after the diagnosis were included in the cohort. The flowchart of data collection is presented in Fig. 1. Data on parturients, their pregnancies (both the one with delivery complicated by an OASI and the subsequent pregnancy) and newborns were extracted manually from the patient records of the parturients by the first author. In addition to the main analysis, we compared the study parturients’ data at the time of the delivery complicated by an OASI to all vaginal deliveries in the study hospital during the same time period. This data for comparison was obtained from the Finnish Perinatal Statistics [1] a publicly available database offering hospital-specific data on selected obstetric interventions as well as maternal and neonatal outcomes.

In the study hospital, the Finnish grip is universally used at the delivery of the fetal head. After delivery, a midwife inspects the parturient’s perineum after every vaginal delivery. If an OASI is suspected, an obstetrician is summoned to examine the lesion. Manual vaginal and rectal assessment is used to diagnose OASI. Follow-up protocol includes assessing anal incontinence symptoms with the Wexner questionnaire [14] six months after delivery. The mode of delivery following the subsequent pregnancy is individually selected based on patient preferences and general obstetric indications.

We compared women with an rOASI to those who had a subsequent delivery without an rOASI. To assess the factors contributing to the choice of delivery mode, data regarding subsequent pregnancies following an OASI were compared between planned vaginal and planned cesarean deliveries and examined the indications for CS. Further, several obstetrical factors (primiparity, maternal overweight and obesity, induction of labor, mode of delivery, episiotomy use, and birthweight > 4,000 g and > 4,500 g) in the delivery primarily complicated by OASI (regarded as the index delivery) were explored in the study cohort and compared to those in all other vaginal deliveries in Tampere University Hospital during the study period to explore risk factors for primary OASI.

### Statistical analyses

Statistical analyses were conducted using SPSS for Windows version 29.0.1.0 (Armonk, NY. IBM Corp). Due to the low incidence of rOASI, data from subsequent deliveries following the one complicated by an OASI are shown in descriptive analyses. Quantitative data were described using medians and minimum and maximum values (as not all data was normally distributed), and categorical variables using percentages. The Mann-Whitney U-test, Chi-squared and Fisher’s exact test were used as appropriate. A *p*-value < 0.05 was considered statistically significant and all *p*-values are two-tailed.

## Results

Between 2009 and 2021, 54,231 parturients had a vaginal delivery in Tampere University Hospital, and 580 (1.07%) of them suffered an OASI. Of these patients, 278 (47.9%) had delivered again in the same hospital by the end of year 2023 and were included in the study cohort.

In the subsequent pregnancy following the delivery complicated by OASI, a majority of parturients (78.1%, *n* = 217) planned a vaginal delivery. Three of them underwent an acute CD whereas 214 had a successful vaginal birth. Of parturients who planned a CD (21.9%, *n* = 61), all but one had a CD. One woman planned a CD, but the labor was very rapid, and she delivered vaginally without perineal trauma. The recurrence of OASI was rare: 98.1% of parturients who delivered vaginally had the subsequent birth without an OASI. Details of subsequent vaginal deliveries with and without rOASI are shown in Table 1. All instances of rOASI were observed in the first delivery after the index pregnancy. In addition, 48 women had at least two deliveries after the index delivery. None of these further deliveries were complicated by an rOASI.

**Table 1:** Descriptive data of subsequent vaginal deliveries after a delivery complicated by obstetric anal sphincter injury (OASI) in Tampere university hospital 2009–2021

	Subsequent vaginal delivery, no OASI *n* = 210		Subsequent vaginal birth, recurrent OASI *n* = 4	
	n or median	% or min, max	n or median	% or min, max
Maternal age, years	32.0	21, 42	28.50	25, 31
BMI	24.2	16.4, 44.8	26.4	24.2, 28.6
Induction of labor	59	21.5	2	50.0
Assisted vaginal delivery	6	2.8	0	0
Presentation other than occiput anterior	29	10.7	0	0
Duration of second stage (min)	12.0	1, 91	9.0	6, 10
Episiotomy	35	12.8	1	25.0
Gestational age at delivery, weeks + days	39 + 3	32 + 2, 42 + 1	40 + 0	39 + 3, 41 + 6
Birth weight (g)	3645	1500, 4975	3807	3475, 4170
Birth weight SD	0.2	−4.0, 3	0.35	−0.7, 1.3
Oxytocin augmentation	176	83.8	3	75

*SD* standard deviation

The four parturients who had an rOASI had spontaneous vaginal deliveries. The duration of the second stage of labor ranged from six to ten minutes and the parturients delivered infants weighing between 3,475 and 4,170 g in occiput anterior presentation.

Post repair procedures were performed for two patients (0.7%) after a primary OASI (none for an rOASI) approximately one year after the injury. In one case the OASI went unnoticed, and only a vaginal and superficial perineal tear repaired immediately postpartum. In the other case, primary repair was performed appropriately after delivery, but the patient suffered from prolonged fecal incontinence. Both women had a CD following their subsequent pregnancy.

In subsequent pregnancies, the most common indication for planning a CD was maternal request or fear of childbirth (85.9%). Parturients who planned a vaginal delivery were slightly younger and taller than those planning a CD, and infants were born at later gestation if a vaginal delivery was planned instead of a CD. Details of subsequent deliveries are shown in Table 2.

**Table 2:** Subsequent deliveries after an obstetric anal sphincter injury (OASI) in Tampere university hospital during 2009–2021

	Subsequent vaginal delivery *n* = 214		Subsequent cesarean delivery *n* = 64		*p*-value
	n or median	% or min, max	n or median	% or min, max	
Maternal age, years	31	21, 42	33	23, 42	0.003
BMI ≥ 30	30	14.2	9	14.1	0.986
Induction of labor	58	27.1			
Estimated weight of fetus, SD^*^	0.18	−3.49, 3.44	0.29	−1.56, 1.94	0.737
Vacuum extraction	6	2.8			
CD by maternal request			55	85.9	
Perineal trauma					
No	172	80.4			
First degree tear	22	10.3			
Second degree tear	16	7.5			
Third degree tear	4	1.9			
Episiotomy	36	16.8			
Gestational age at delivery, weeks + days	39 + 5	32 + 6, 42 + 1	39 + 1	32 + 2, 39 + 6	< 0.001
Birth weight, g	3673	1500, 4975	3495	1673, 4625	0.228
Birth weight SD	0.2	−4, 3	−0.1	−1.7, 3.0	0.787
Birth weight > 4.5 kg	5	2.3	3	4.7	0.391

*SD* standard deviation, *CD* cesarean delivery

^*^Ultrasound weight estimation performed 10 days before birth, data available for 130 (46.8%) parturients

Parturients were more likely to have a CD if the preceding delivery (complicated by an OASI, referred to as index delivery) had been postterm, labor had been induced, presentation had been other than occiput anterior, the second stage had been prolonged, and if an episiotomy had been performed. More than half of those who had a CD in the subsequent birth had had assisted vaginal delivery at the index delivery. Compared to all other vaginal deliveries in the study hospital during the same time period, assisted vaginal delivery, birthweight exceeding 4,000 g or 4,500 g, an episiotomy, and postterm pregnancy were more common in deliveries complicated by OASI. See Table 3 for details of the index pregnancies.

**Table 3:** All vaginal deliveries in Tampere university hospital during 2009–2021. Index deliveries of women who had an obstetric anal sphincter injury (OASI) and later had another delivery at the study hospital is compared between groups according to the delivery mode in the subsequent delivery, and to all other vaginal deliveries during the study period. Table 3 describes the preceding deliveries complicated by an OASI

	Birth with OASI - subsequent vaginal birth (sVD) *n* = 214 (77.0%)		Birth with OASI - subsequent cesarean delivery (sCD) *n* = 64 (23.0%)		*p*-value sVD vs. sCD	All other vaginal deliveries (oVD) *n* = 53 953		*p*-value sVD vs. oVD
	n or median	% or min, max	n or median	% or min, max		n	%	
Primiparous	199	93.0	59	92.2	0.787^*^	22 186	41.1	< 0.001
- VBAC^**^	5	2.3	3	4.7	0.391^*^			
- Multipara^**^	10	4.7	2	3.1	0.739^*^			
BMI ≥ 30	24	11.4	5	7.8	0.416	7056	13.1	0.332
BMI ≥ 25	75	36.2	19	29.7	0.336	12 229	22.7	< 0.001
Induction of labor	43	20.1	17	26.6	0.270	13 569	25.1	0.227
Spontaneous vaginal delivery	145	67.8	31	48.4	0.005	49 298	91.4	< 0.001
Assisted vaginal delivery	69	32.2	33	51.6	0.005	4655	8.6	< 0.001
Episiotomy	115	53.7	48	75.0	0.002	14 008	26.0	< 0.001
Presentation other than occiput anterior	32	15.0	15	23.4	0.112			
Birthweight SD	0.1	−2, 3	0.2	−2, 4	0.425			
Birthweight ≥ 4 kg	52	24.3	18	28.1	0.536	9467	17.5	0.003
Birthweight ≥ 4,5 kg	10	4.7	3	4.7	1	1298	2.4	0.049
Gestational age ≥ 42 + 0 weeks at birth	13	6.1	8	12.5	0.106	1754	3.2	< 0.001
Duration of second stage, minutes	31	1, 111	51	4, 95	< 0.001			
Second stage > 60 min	39	18.2	28	43.8	< 0.001			
OASI degree^***^					< 0.001			
- < 50%	132	61.7	24	37.5				
- > 50%	80	37.4	34	53.1				
- 4th degree tear	2	0.93	6	9.4				

^*^Fisher’s Exact

^**^VBAC describes multiparous parturients with no previous vaginal deliveries; Multipara describes parturients who have had at least one vaginal delivery before the delivery complicated by OASI. Nine of these were secundiparous and three had had two vaginal deliveries before OASI

^***^proportion of external sphincter torn; in fourth degree tears also the intestinal mucosa was damaged

Incontinence symptoms were routinely assessed using the Wexner questionnaire six months after an OASI. Regarding index delivery, the survey results of 117 mothers (42%; from 2012 onwards) were available and analyzed. Of these, 29 patients (24.8%) reported perfect continence (Wexner score 0), and 100 (85.5%) reported a score < 4. Vaginal delivery following the subsequent pregnancy was more common if the parturient had recovered well: 89.7% of perfectly continent women had a vaginal delivery compared to 52.9% of the 17 women who had scored 4 or more on the Wexner questionnaire (*p* = 0.005).

## Discussion

In our study of 278 deliveries after a preceding OASI, the risk of rOASI was low, at 1.9% of attempted vaginal deliveries, comparable to the risk of OASI in primiparous birth at 1.4% in the Finnish population and the overall risk of 1.07% in our population. Due to low incidence, no risk factors for rOASI could be identified. In subsequent pregnancies, most parturients planned and accomplished vaginal delivery. Maternal request was the most common indication for planning a cesarean delivery in subsequent pregnancy. Parturients who planned a CD for their subsequent delivery had more often had an assisted vaginal delivery and a prolonged second stage in their preceding deliveries.

The risk of OASI in the subsequent birth has been reported to be significantly higher in parturients who suffered an OASI at the previous birth compared to parturients without a previous OASI. In a British study, 10.2% of parturients with a preceding OASI had an rOASI in the subsequent birth, whereas among all multiparas the incidence was only 1.2% [11]. Further, a register-based study comparing numbers from 1997 to 2002 in Finland, Sweden and Norway reported an OASI recurrence rate of 2.1%, 4.5% and 6.6%, clearly higher than the incidence of OASI in multiparous parturients without a preceding sphincter injury (0.8%, 0.7% and 1.7%, respectively). The relatively low recurrence rate in Finland found in this study can partly be explained by high CD rates in subsequent births (more than 50%), while in Norway and Sweden the CD percentage was around 16% [13]. In our study, the incidence of rOASI was low though the CD rate was more modest, possibly related to the different time period or to the strong tradition of vaginal delivery in our hospital. The midwives’ technique of supporting the perineum during delivery appears to provide effective protection against perineal tears, even in subsequent births. If the parturient desires vaginal delivery and there are no obstetric contraindications, a trial of vaginal delivery can be recommended.

Primiparity strongly predisposes parturients to perineal trauma [15]. In our study, the rate of rOASI was comparable to the OASI rate of primiparous births. A Canadian study reported similar findings with the rate of both OASI in primiparous women and rOASI at 5.3% [16]. A cesarean section minimizes the risk of rOASI but carries other risks for both parturient and infant [17–22]. With such a low risk of rOASI, a vaginal delivery could be recommended to most patients.

A systematic review assessed risk factors for rOASI, pooling very diverse populations in eight individual studies. The incidence of primary OASI ranged from 0.6 to 19.3% and rOASI from 2.0 to 13.4%. Operative vaginal delivery, a preceding fourth degree tear, advanced maternal age and increasing infant birthweight were shown to increase the risk of rOASI [2]. Regarding the absolute risk of rOASI, this review provides a global estimate of average risk, and in low risk settings the risk estimate per se is not applicable for patient counseling and shared decision-making. Another review and meta-analysis studying risk factors did not show an association between preceding OASI severity and infant birthweight but recognized increasing gestational age, occiput posterior presentation, operative vaginal delivery and oxytocin augmentation as predisposing parturients to OASI recurrence [22]. In our study, all deliveries complicated by rOASI were spontaneous vaginal deliveries of normal-sized neonates, demonstrating the difficulty in predicting rOASI.

The incidence of OASI has also been slightly increasing in Finland over the last few decades [1]. The increase of ruptures has been associated with improved diagnostics but also with an increase in several other factors predisposing parturients to OASI, such as obesity and advanced maternal age [23]. From a global perspective, preventing the primary OASI is of paramount importance given the increased morbidity as well as increased CD rates in subsequent deliveries, predisposing patients to further complications. Previous studies have shown encouraging results in decreasing the rate of tears by training staff to prevent OASI [7, 23–25].

The weakness of this study is its quite small study population, limited by the low incidence of OASI and especially that of recurrent injury. However, studies from other countries are not valid in settings with very low incidence of OASI for the same reason, and these results can directly be used in clinical settings to help parturients make informed choices on mode of delivery in subsequent pregnancy after OASI. Given the observational nature of the study, we were reliant on data that had been collected primarily for clinical, rather than scientific, purposes– for instance, the Wexner scores. The study population was also limited due to including patients from only one hospital, but this design ensured very reliable data collection directly from patient records and uniform management of deliveries. Overall, the quality of midwifery care in Finland is uniformly high, with a low incidence of severe perineal trauma. While rOASI are not routinely documented, the rate of primary OASI is consistently similar throughout the country [1].

A complicated birth is a risk factor for a negative birth experience [26] and some parturients will choose a CD as the method of delivery for the subsequent birth. Women who experienced an OASI also wish to postpone or even abandon subsequent pregnancy more often compared to women who did not suffer an OASI [27]. In our study, factors that are associated with a negative birth experience, such as assisted vaginal delivery, an episiotomy, and a long second stage, were overrepresented in the index deliveries of women choosing a CD for the subsequent birth. It is possible, when a symptomatic woman expresses a preference for CD, the discussion regarding the mode of delivery may be less comprehensive than with women who are initially positively inclined toward vaginal delivery. Vaginal delivery was achieved in 89.7% of women without any fecal incontinence, compared to 52.9% of those with a Wexner score 4 or higher. Based on the available Wexner scores, lingering symptoms may lead to a preference for CD. This mirrors another study reporting that women who expressed that a previous OASI still impacted their life were significantly more likely to have a cesarean section in the subsequent birth [28]. Evidence regarding the association between tear severity and long-term complications is conflicting [29–32], but some professionals have recommended a CD after a fourth degree tear [33].

## Data Availability

The authors confirm that access restrictions apply to the data. The GDPR legislation requires us to protect the identity of participants, and the raw data cannot be publicly shared. Hospital-specific (Tampere University Hospital) data on several obstetric and neonatal factors by mode of delivery is publicly available from the Finnish Perinatal Statistics.

## Abbreviations

OASI: Obstetrical anal sphincter injury
rOASI: Recurrent obstetrical anal sphincter injury
CD: Cesarean delivery
